# The Maximal Oxygen Uptake Verification Phase: a Light at the End of the Tunnel?

**DOI:** 10.1186/s40798-017-0112-1

**Published:** 2017-12-08

**Authors:** Gustavo Z. Schaun

**Affiliations:** Neuromuscular Assessment Laboratory, Physical Education School, Federal University of Pelotas, Rua Luís de Camões, 625, Tablada, Pelotas, RS 96055-630 Brazil

**Keywords:** Exercise, Oxygen consumption, Incremental exercise, Graded exercise test, Cardiopulmonary exercise testing, Plateau, Criteria

## Abstract

Commonly performed during an incremental test to exhaustion, maximal oxygen uptake (V̇O_2max_) assessment has become a recurring practice in clinical and experimental settings. To validate the test, several criteria were proposed. In this context, the plateau in oxygen uptake (V̇O_2_) is inconsistent in its frequency, reducing its usefulness as a robust method to determine “true” V̇O_2max_. Moreover, secondary criteria previously suggested, such as expiratory exchange ratios or percentages of maximal heart rate, are highly dependent on protocol design and often are achieved at V̇O_2_ percentages well below V̇O_2max_. Thus, an alternative method termed verification phase was proposed. Currently, it is clear that the verification phase can be a practical and sensitive method to confirm V̇O_2max_; however, procedures to conduct it are not standardized across the literature and no previous research tried to summarize how it has been employed. Therefore, in this review the knowledge on the verification phase was updated, while suggestions on how it can be performed (e.g. intensity, duration, recovery) were provided according to population and protocol design. Future studies should focus to identify a verification protocol feasible for different populations and to compare square-wave and multistage verification phases. Additionally, studies assessing verification phases in different patient populations are still warranted.

**Electronic supplementary material:**

The online version of this article (10.1186/s40798-017-0112-1) contains supplementary material, which is available to authorized users.

## Key Points

➢ Both supramaximal and submaximal protocols appear to be suitable for measuring maximal oxygen consumption (V̇O_2max_), while verification phase durations between 2 and 3 min should already be adequate to confirm V̇O_2max_ for most, but not all, subjects.

➢ Based on available data, recovery time between phases does not appear to be critical, although more research is warranted, and processing data with 15- to 30-s time averages seems reasonable.

➢ When all safety measures are taken, the verification phase seems to be well tolerated in both healthy and patient populations in general. Nevertheless, more research is still necessary to further confirm verification phase applicability in older adults and clinical populations, as well as in longitudinal studies.

## Review

### Background

Since its identification in the 1920s, the maximal oxygen uptake (V̇O_2max_) has gained substantial clinic and scientific relevance. Initially proposed by Hill and Lupton [1], V̇O_2max_ represents a physiological ceiling on the capacity to increase alveolar O_2_ uptake, O_2_ transport and/or consumption at tissue level in response to an increase in workload and metabolic demand. Currently, it is accepted as the gold standard to assess cardiorespiratory fitness [2] and is also one of the best individual predictors of all-cause mortality and cardiovascular disease risk [3–5]. Consequently, a sufficiently high V̇O_2max_ is important to reduce these risks and for maintenance of quality of life in the general population.

V̇O_2max_ has also become relevant within the field of exercise performance. In endurance sports such as running, cycling and cross-country skiing, for example, a high V̇O_2max_ is a conditioning variable for sporting success [6]. In other sports, like football and rugby, high cardiorespiratory fitness may assist recovery between the brief high-intensity efforts, characteristic of these modalities [7, 8]. Moreover, several physiologists and conditioning coaches use variables associated with V̇O_2max_ (e.g. velocity, power, heart rate) to design and prescribe training programs. The efficiency of these programs to enhance cardiorespiratory fitness can also be determined by V̇O_2max_ reassessment.

Accordingly, valid and reliable tests (i.e. capable of being replicated) are necessary to proper evaluate V̇O_2max_. Over the years, the most commonly used protocol is the incremental load test performed as a progressive ramp or from step increments until exhaustion [9]. Protocol characteristics, such as the initial velocity/power and incline, workload increments, stage duration and ergometer, are designed according to the population being assessed to allow V̇O_2max_ to be properly achieved. However, a question that continues to intrigue researchers and scientists for decades is whether, in fact, it is possible to attest that the V̇O_2max_ measured is a real maximal value. For this purpose, the plateau in V̇O_2_ stands out as the main criterion employed [10–12]. As will be discussed in an appropriate session, a plateau is not always identified in all tested individuals. Hence, several secondary criteria were proposed [13], which have been extensively criticized in recent years for their lack of validity and sensitivity [2, 10–12, 14–17].

Thereafter, an alternative solution termed “verification phase” was proposed [18]. In summary, after the incremental test, a new effort is performed and V̇O_2max_ results are compared between phases. The validity of this procedure has already been confirmed in eutrophic and obese adults, patient with clinical conditions and children [12]. Midgley and colleagues, in 2007, published an article in *Sports Medicine* suggesting that future studies should focus on investigating the verification phase protocols and how to improve their utility and validity [14]. Approximately 10 years later, it is important to assess scientific findings that have followed regarding the verification phase and whether or not there are issues that still require further attention. Thus, the aim of the present review was to provide an update on the knowledge produced on the $\dot{V}{\mathrm{O}}_{2\max }$ verification phase, highlighting the advances in methodology and suggesting future directions for research. Furthermore, although previous research has confirmed the usefulness of this phase and has clarified the rationale for its incorporation [12], no investigation tried to summarize how it has been done while also providing clear recommendations on how the verification phase should be performed as a whole (e.g. intensity, duration, protocol design, test validation). Hence, the present paper should assist researchers, as well as professionals, to employ the verification phase more adequately. Due to the great heterogeneity between protocols and among studied populations, the author considered it inappropriate to perform a systematic review and meta-analysis.

### V̇O_2_ Plateau—Fool’s Gold?

The notion of a reduction in the O_2_ uptake slope near the end of the incremental test, despite a progressive increase in intensity, also seems to have been introduced by Hill and his colleagues [1, 19], although this is not a consensus view [20]. Regardless, this plateau in oxygen uptake (V̇O_2_) is understood as the best evidence that a “true” V̇O_2max_ was reached during the incremental test [9]. However, more than 80 years have passed since Hill’s pioneering studies and the answer to which would be the definitive criterion to confirm the occurrence of a plateau has not yet been given. Taylor et al. have possibly suggested what came to be the most popular criterion in the recent decades for this purpose [21]. These authors concluded that changes ≤ 150 ml min^−1^ between two consecutive stages meant that it could “[...] safely be assumed that a maximal oxygen uptake [...]” had “[...] been attained”, that is, values ≤ 50% of the expected increase. On this regard, Taylor and colleagues were able to demonstrate a plateau in 108 out of 115 tests (i.e. 94%). However, the 150 ml min^−1^ threshold has been criticized because of its lack of theoretical and statistical basis, as well as its lack of specificity in relation to the protocols currently used [22]. Investigations that attempted to replicate Taylor’s findings found a great variability in the percentage of subjects who presented a plateau in V̇O_2_ [23, 24].

Moreover, other threshold values have also been adopted, such as ≤ 100 ml kg^−1^ or ≤ 50 ml kg^−1^ of O_2_ [22, 25] making it even more difficult to compare studies. Regardless of the threshold adopted, a strong inconsistency as to the number or percentage of plateaus observed is found in the literature [20, 26]. Previous studies have identified 100% [22] and 94% [21] plateau incidence, while others reported values as low as 47%, 24% [27], 17% [28] and even 0% [29]. Beltz et al. [2] emphasize that age, modality tested and how data are processed are among the main factors that can influence the incidence of plateaus (for a detailed review on data processing, see Robergs et al. [30]). Physical fitness and incremental test protocol are factors that can also influence the incidence of plateaus in V̇O_2_ [11, 31]. As stated by Midgley and Carroll [11], together, these considerations reduce the usefulness of the plateau criterion as a robust method for determining a “true” V̇O_2max_, not because of the plateau itself, but by the methodology used in its identification.

At present, there seems to be a consensus in the literature regarding the existence of the plateau phenomenon in V̇O_2_ [20, 22, 26]. Nevertheless, some recent studies have questioned the necessity of this phenomenon to characterize that a V̇O_2max_ has been reached [15, 26, 28]. After submitting 52 well-trained distance runners to an incremental treadmill test and a second bout to exhaustion at an intensity 30% higher than the incremental phase, Hawkins and colleagues [32] found no difference between V̇O_2max_ in both tests. Similarly, eight well-trained men underwent two maximal and one supramaximal test on a combined arm and leg exercise [33]. No differences were observed in V̇O_2max_, contributing to the notion that actually there is a limit on V̇O_2_ (see also Day et al. [28]). Furthermore, it should be noted that the same subject who performs an incremental test twice can show a plateau in only one of them while presenting similar V̇O_2max_ values between the tests [15, 34, 35]. Therefore, although the plateau is still considered the best evidence to confirm that a V̇O_2max_ has been reached, is the effort employed to identify this phenomenon worth it considering its inconsistency?

### Secondary Criteria

Based on these assumptions, researchers deemed necessary to define secondary criteria for when a V̇O_2_ plateau was not evident [36]. The most common ones are thresholds in the respiratory exchange ratio (RER); age-predicted maximal heart rate (HR_max_); blood lactate concentration; and on Borg’s rating of perceived effort scale (RPE) [13, 17, 24, 36]. The rationale is that in the absence of an evident plateau, researchers could attest that a maximal effort was given [36, 37]. However, some subjects do not reach the criteria even when a maximum effort was given. On the other hand, these criteria also often end up including subjects who did not perform maximally and, consequently, underestimates V̇O_2max_. Poole et al. [17] showed in eight healthy men that more liberal thresholds, such as ± 10 bpm of the age-predicted HR_max_ and RER ≥ 1.10, were already reached at 75 to 80% of the V̇O_2max_. Experimental studies employing these criteria may underestimate V̇O_2max_ during baseline testing and, in turn, overestimate V̇O_2max_ increase after the intervention, for example.

Finally, and perhaps more importantly, contrary to what would be expected, these criteria are also not able to differentiate those who demonstrate a plateau in V̇O_2_ from those who do not [17, 31]. After an incremental test on a cycle ergometer, only two out of 99 adults presented a plateau in V̇O_2_, RER ≥ 1.15 and reached ± 10 bpm of the age-predicted HR_max_ [16]. In addition, these cutoff points can be decided a posteriori, that is, researchers can choose the most convenient value based on the results obtained. These considerations led previous authors to suggest the complete rejection of secondary criteria to validate maximal tests [11, 17]. Yet, in 2010, a survey encompassing approximately 75 subjects trained in the field of exercise physiology showed that 52% performed their V̇O_2max_ assessments and data processing based on subjective concepts such as “beliefs and traditions” [30]. Therefore, greater efforts are needed to identify alternative methods and to popularize these alternatives in the clinical and scientific community. Only then will inadequate criteria cease to be employed.

### Verification Phase: a Light at the End of the Tunnel?

So far, we have seen that the incidence of a plateau in V̇O_2_ during incremental tests is or may be low. Additionally, the secondary criteria used, at least those mentioned above, present great inter-subject variability compromising the sensitivity and reliability of these criteria (see the “Secondary Criteria” section). As a consequence, a viable alternative to confirm true V̇O_2max_ still need to be identified. Dating from at least 1982, based on a book chapter written by Thoden and colleagues, the “exhaustive phase”, which would later be renamed to “verification phase” [18], proposed a second effort at an intensity higher than the incremental test to be performed [2, 11]. The first scientific report on the use of verification phases seems to be that of Niemelä et al. [16]. The authors assessed 16 healthy men who performed an incremental ramp test on a cycle ergometer and 1 week later underwent a warm-up comprising one or two submaximal workloads followed by supramaximal exertion. Additionally, Morgan et al.’s [38] study was suggested as the primary study to incorporate the verification phase as part of its methods [11]. Highly trained runners completed a maximal treadmill test and those who did not show a plateau in V̇O_2_ during the test performed a four-minute supramaximal effort, 10 min after the incremental test. Both studies found no differences between the means in V̇O_2max_ from the incremental test and the verification phase. Although there are some caveats regarding the methodology and data reporting of these studies, it is undeniable that both provided promising results and demonstrated that the use of the verification phase could be a viable method to confirm V̇O_2max_ (for a historical perspective on the verification phase, readers are referred to Beltz et al. [2] and Midgley and Carroll [11]).

**Fig. 1:**
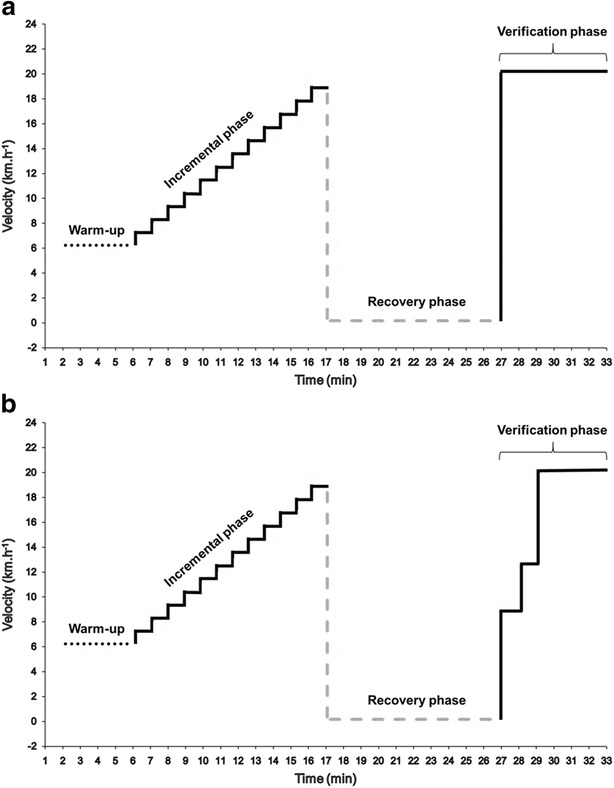
Schematic illustration of an incremental test followed by either a square-wave (**a**) or a multistage (**b**) verification phase. Incremental protocol consists of a 5-min warm-up followed by 1 km h^−1^ increments every minute until exhaustion (i.e. 19 km h^−1^). After 10 min of passive recovery, an effort at one stage higher (20 km h^−1^) than the last stage completed during the incremental phase is performed directly (**a**) or preceded by a “warm-up” corresponding to 2 min at 50% and 1 min at 70% of the maximal velocity reached during the incremental phase (**b**)

Therefore, it is important to define what exactly is configured as a verification phase. According to Pettit et al. [39], it is an exhaustive square-wave effort used to corroborate V̇O_2max_ measured during incremental testing. For Midgley et al. [35], the verification phase is characterized as a recovery of 5–15 min after the completion of the progressive test followed by an effort to exhaustion one stage higher than the progressive test. However, there are studies that perform the verification phase on a separate day [40] and at intensities lower than maximal [29, 41, 42]. Moreover, some use a square-wave design while others use a multistage approach (Fig. 1). Thus, a more comprehensive definition encompassing these characteristics becomes necessary. In a broad sense, the verification phase is an effort performed after an incremental test to exhaustion, in the same session or not, with intensities ranging from submaximal (above critical power) to supramaximal that allow sufficient time for V̇O_2max_ to be reached. The V̇O_2max_ measured during the verification phase is then compared to that of the incremental phase and if both do not differ, based on a given criterion, the test is considered valid and V̇O_2max_ as true.

Although the verification phase has been suggested and investigated for almost 30 years, not many studies applied this method in their investigation [37]. It was suggested that the reason for this would be the lack of studies supporting its validity [11]. However, in recent years several experiments have been carried out showing that the verification phase can confirm true V̇O_2max_ in different populations ranging from highly trained athletes [32, 43], recreationally trained subjects [41, 42], physically active [44], sedentary [31] and clinical populations [45, 46]. These studies along with their protocols are presented in Table 1. Accordingly, the next sections will seek to summarize how the verification phase has been performed and provide suggestions for its implementation whenever possible.

**Table 1:** Characteristics of previous studies that employed the verification phase to confirm V̇O_2max_ attained during an incremental test to exhaustion

Reference	Incremental phase	Recovery phase	Verification phase	Verification during the same session?	V̇O_2max_ during incremental phase (ml min^−1^)	V̇O_2max_ during verification phase (ml min^−1^)
Niemelä et al. [16]	Continuous test on a cycle ergometer (1 min stages)	NA	Supramaximal workload based on highest V̇O_2_ obtained during incremental test (NSW)	Separate day	2950 (590)	3050 (490)
Stachenfeld et al. [67]	Continuous test on a cycle ergometer (1 min stages)	NA	115% of peak workload attained during incremental phase. If necessary, a second phase at 125% was performed (NSW).	Separate day	3280 (−)	3350 (920)
Day et al. [28]	Ramp incremental test on a cycle ergometer	NA	90% of the peak power output attained in the incremental test	Separate day	3640 (700)	3640 (700)
Midgley et al*.* [35]	Continuous test on a treadmill (1 min stages)	10 min active	0.5 km/h higher than the last incremental stage completed	Yes	4041 (455)	3994 (447)
	Exact same test	10 min active	Exact same test	Yes	4010 (379)	4029 (432)
Rossiter et al. [29]	Ramp incremental test on a cycle ergometer.	5 min active	95% of the peak power output attained in the incremental test.	Yes	4105 (478)	4117 (528)
	Ramp incremental test on a cycle ergometer.	5 min active	105% of the peak power output attained in the incremental test.	Yes	4149 (502)	4090 (446)
Midgley et al. [37]	Continuous test on a treadmill. (1 min stages).	5 min passive	0.5 km/h higher than the last incremental stage completed.	Yes	4093 (538)	4068 (531)
	Discontinuous test on a treadmill (2 min stages and 30 s between)	5 min passive	1 km/h higher than the last incremental stage completed.	Yes	4096 (516)	4075 (522)
	Discontinuous test on a treadmill (3 min stages and 30 s between)	5 min passive	1 km/h higher than the last incremental stage completed	Yes	3980 (488)	4071 (531)
Hawkins et al. [32]	Continuous test on a treadmill (2 min stages)	NA	Work load correspondent to ≥ 130% V̇O_2max_	Separate day	63.3 (6.3) ml kg^−1^ min^−1^	62.9 (6.2) ml kg^−1^ min^−1^
Foster et al*.* [51]	Continuous test on a cycle ergometer (1 min stages)	1 min active	25 W higher than peak power output attained in the incremental test	Yes	3950 (750)	4060 (750)
	Continuous test on a treadmill (3 min stages)	3 min active	0.8 km/h (women) and 1.6 km/h (men) higher than the last incremental test stage completed	Yes	4090 (970)	4030 (1160)
Poole et al*.* [17]	Ramp incremental test on a cycle ergometer	NA	105% of the peak power output attained in the incremental test	Separate day	4030 (100)	3950 (110)
Astorino et al*.* [31]	Ramp incremental test on a cycle ergometer	NA	105% of the peak power output attained in the incremental test	Separate day	2370 (690)	2290 (750)
	Continuous test on a treadmill (20 s stages)	60–90 min passive	115% of the peak power output attained in the incremental test	Yes	2670 (600)	2750 (760)
Midgley et al. [36]	Continuous test on a cycle ergometer (1 min stages)	10 min passive	One stage higher than the last stage attained in the incremental test (NSW)	Yes	4054 (467)	3958 (381)
	Continuous test on a treadmill (1 min stages)	10 min passive	One stage higher than the last stage attained in the incremental test (NSW)	Yes	3863 (394)	3915 (466)
Astorino and White [61]	Continuous test on a cycle ergometer (1 min stages)	10 min active	One stage higher than the last stage attained in the incremental test	Yes	2900 (600)	2900 (600)
Wood et al. [68]	Continuous test on a treadmill (1 min stages)	5–10 min passive	0.5 km/h within the last incremental stage completed (NSW)	Yes	34.5 (7.4)ml kg^−1^ min^−1^	Not reported
Kirkeberg et al. [41]	Continuous test on a treadmill (1 min stages)	3 min active	Two stages lower than the last stage attained in the incremental test	Yes	49.24 (5.31) ml kg^−1^ min^−1^	48.97 (5.95) ml kg^−1^ min^−1^
	Exact same test	3 min active	Two stages lower than the last stage attained in the incremental test	Yes	48.90 (5.08) ml kg^−1^ min^−1^	47.45 (4.48) ml kg^−1^ min^−1^
	Exact same test	3 min active	Two stages lower than the last stage attained in the incremental test	Yes	49.07 (4.70) ml kg^−1^ min^−1^	48.44 (5.02) ml kg^−1^ min^−1^
Scharhag-Rosenberger et al*.* [40]	Discontinuous test on a treadmill (3 min stages and 20 s between)	10 min passive	110% of the velocity attained in the incremental test. If necessary, new verification phase at 115% was performed (NSW).	Yes	3824 (988)	3722 (991)
	Discontinuous test on a treadmill (3 min stages and 20 s between)	10 min passive	110% of the velocity attained in the incremental test. If necessary, new verification phase at 115% was performed (NSW).	Separate day	3824 (988)	3752 (995)
Bowen et al. [64]	Ramp incremental test on a cycle ergometer	5 min active	95% of the peak power output attained in the incremental test	Yes	14.5 (3.0)ml kg^−1^ min^−1^	14.7 (3.1) ml kg^−1^ min^−1^
Beltrami et al*.* [69]	Continuous test on a treadmill (1 min stages)	15 min passive/active	One stage higher than the last stage attained in the incremental test (NSW)	Yes	61.3 (7.8) ml kg^−1^ min^−1^	61.2 (5.5) ml kg^−1^ min^−1^
	Continuous test on a treadmill (1 min stages)	15 min passive/active	One stage higher than the last stage attained in the incremental test (NSW)	Yes	61.2 (4.8) ml/kg/min	60.9 (3.5) ml kg^−1^ min^−1^
Leicht et al. [52]	Wheelchair continuous test on a treadmill (1 min stages)	2 min passive + 5 min active	100% of the velocity attained in the incremental test. However, incline was increased by 0.6%.	Yes	1574 (354)	1649 (393)
	Wheelchair continuous test on a treadmill (1 min stages)	2 min passive + 5 min active	100% of the velocity attained in the incremental test. However, incline was increased by 0.6%.	Yes	2470 (335)	2395 (335)
	Continuous test on a treadmill (40 s stages).	2 min passive + 5 min active	100% of the velocity attained in the incremental test. However, incline was increased by 0.3%.	Yes	3059 (627)	2978 (530)
Mier et al. [24]	Continuous test on a treadmill (2 min stages).	10 min active	One stage higher than the last stage attained in the incremental test (NSW)	Yes	53.6 (5,6)	55.5 (5,6)
Dalleck et al. [50]	Continuous test on a cycle ergometer (20 s stages for men, 30 s for women)	5 min active + 60 min passive	105% of the peak power output attained in the incremental test	Yes	2329 (762)	2309 (760)
Saynor et al*.* [45]	Ramp incremental test on a cycle ergometer	5 min active + 10 min passive	110% of the peak power output attained in the incremental test (NSW)	Yes	1770 (570)	Not reported
Sedgeman et al. [42]	Continuous test on a cycle ergometer (1 min stages)	3 min active	Two stages lower than the last stage attained in the incremental test	Yes	49.81 (5.49) ml kg^−1^ min^−1^	49.93 (6.63) ml kg^−1^ min^−1^
	Continuous test on a cycle ergometer (1 min stages)	3 min active	105% of the peak power output attained in the incremental test	Yes	50.10 (6.83) ml kg^−1^ min^−1^	49.19 (6.74) ml kg^−1^ min^−1^
Nolan et al. [44]	Continuous test on a treadmill (1 min stages)	20 min passive	105% of the work load achieved during the incremental test	Yes	56.9 (9.6)ml kg^−1^ min^−1^	57.2 (9.0) ml kg^−1^ min^−1^
	Continuous test on a treadmill (1 min stages)	60 min passive	105% of the work load achieved during the incremental test	Yes	56.2 (9.0) ml kg^−1^ min^−1^	56.2 (9.1) ml kg^−1^ min^−1^
	Continuous test on a treadmill (1 min stages)	20 min passive	115% of the work load achieved during the incremental test	Yes	57.5 (9.2) ml kg^−1^ min^−1^	56.6 (9.6) ml kg^−1^ min^−1^
	Continuous test on a treadmill (1 min stages)	60 min passive	115% of the work load achieved during the incremental test	Yes	57.1 (8.4)ml kg^−1^ min^−1^	56.0 (9.6) ml kg^−1^ min^−1^
Straub et al. [48]	Ramp incremental test on a cycle ergometer	10 min passive	110% of the velocity attained in the incremental test (NSW)	Yes	3840 (730)	3840 (680)
Sawyer et al. [53]	Ramp incremental test on a cycle ergometer	5–10 min active	100% of the peak power output attained in the incremental test.	Yes	2290 (710)	2340 (670)
Scheadler and Devor [70]	Continuous test on a treadmill (1 min stages)	NA	Supramaximal work load determined individually (~ 10.2% greater than V̇O_2max_; NSW).	Separate day	64.9 (8.2)ml kg^−1^ min^−1^	Not reported
Colakoglu et al. [71]	Continuous test on a cycle ergometer (4, 2 and 1 min stages)	NA	100% of the work load achieved during the incremental test. If necessary, new verification phases at 105, 110 and 115% were performed (NSW).	Separate day	4110 (690)	4560 (600)*
Taylor et al. [72]	Continuous test on a treadmill (1 min stages)	15 min passive/active	One stage higher than the last stage attained in the incremental test (NSW)	Yes	4220 (1050)	3880 (880)
	Continuous test on a treadmill (1 min stages)	15 min passive/active	One stage higher than the last stage attained in the incremental test (NSW)	Yes	4080 (860)	3880 (870)
Weatherwax et al. [43]	Continuous test on a treadmill (15 s stages for men, 20 s for women)	20 min passive	0.48 km/h (women) and 0.64 km/h (men) higher than the last incremental test stage completed (NSW)	Yes	3653 (656)	3621 (629)*
Bhammar et al. [49]	Continuous test on a cycle ergometer (1 min stages)	15 min passive	105% of the work load achieved during the incremental test (NSW)	Yes	1570 (270)	1710 (310)*
	Exact same test	15 min passive	Exact same test	Yes	1840 (480)	1940 (470)*

*V̇O*_*2*_ oxygen uptake, *V̇O*_*2max*_ maximal oxygen uptake, *NA* non-applicable, *NSW* not square-wave (i.e. verification phase was multistage)

*A significant difference (*P* < 0.05) between incremental phase and verification phase V̇O_2max_, as reported by the authors. Note: whenever possible, authors were contacted to provide unavailable data

### How It Should Be Done—Is There Consensus?

#### Intensity

As stated in the previous section, intensities employed during the verification phase ranged from submaximal to supramaximal efforts. Specifically, intensities between 90 and 130% of those associated with $\dot{\mathrm{V}}{\mathrm{O}}_{2\max}\left(\mathrm{i}\dot{V}{\mathrm{O}}_{2\max}\right)$ have already been used (Table 1), and there is still no consensus as to the correct option, if there is one. According to some authors, maximal and submaximal intensities would not incorporate the original plateau concept, indicated by the absence of increment in V̇O_2_ versus an increase in exercise intensity [14]. On the other hand, it seems that exercise intensities above the critical power would already be enough to evoke V̇O_2max_ [39] provided that exercise duration is sufficient. Day et al. [28] submitted 71 healthy men to a verification phase at 90% iV̇O_2max_ on a cycle ergometer and found no difference in the mean V̇O_2max_ between incremental and verification phases. Similarly, Kirkeberg et al. [41] assessed 12 recreationally trained men at two stages previous to that reached at the end of the incremental test, and no difference in V̇O_2max_ was found as well. More recently, one study attempted to compare distinct verification phases, one at 105% of the peak work rate and another at two stages prior to the end of the incremental test [42]. The authors concluded that both verification phases appeared to be valid and no differences were found between them. Comparable results were also reported by Rossiter et al. [29] between verification phases at 105 and 95% of the peak work rate.

Nonetheless, some aspects can be observed in order to select an appropriate intensity. Steeper incremental protocols and short duration stages (e.g. 1 min) tend to result in higher peak workloads at the end of the test, while less steep protocols or longer stages (e.g. 3 min) terminate at workloads closer to critical power [39, 47]. Considering that the verification phase is usually performed based on the peak velocity or power attained at the incremental test, protocols with longer or less steep stages followed by submaximal verification phases could end up using intensities below the critical power for some subjects. Conversely, shorter or steeper stages could result in very high intensities and very short verification phases, making it impossible for certain subjects to reach V̇O_2max_ before exhaustion [2, 11]. This led some authors to suggest that incremental tests encompassing shorter stages and longer stages were confirmed by submaximal and supramaximal intensities, respectively [39]. However, in general both supramaximal and submaximal protocols appear to be able to confirm V̇O_2max_ on most occasions to a greater degree than other criteria [17, 28, 29, 40, 41, 48] (readers are also referred to Additional file 1: Table S1).

**Fig. 2:**
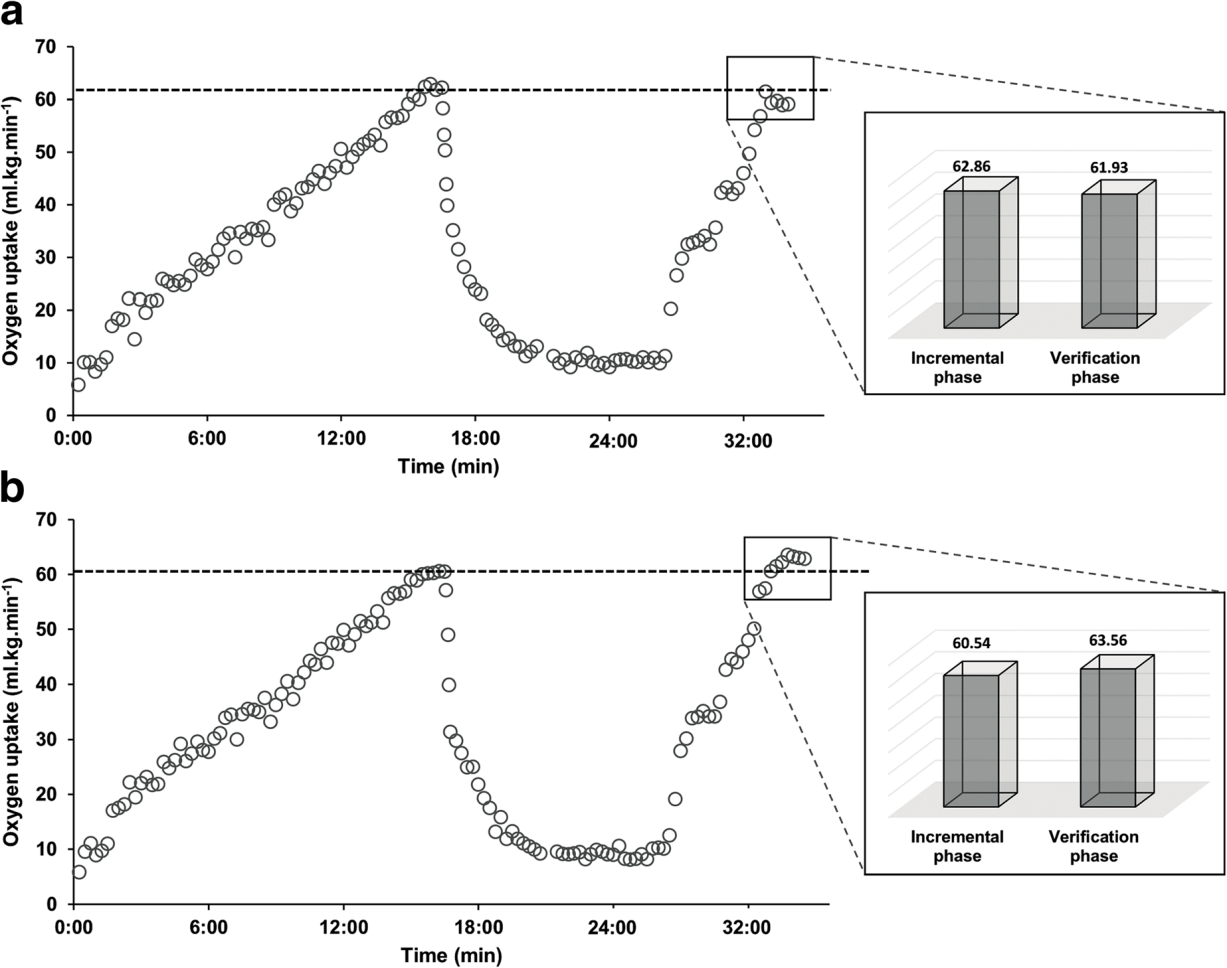
Oxygen uptake (V̇O_2_) responses to an incremental test followed by a multistage verification phase for two representative subjects. **a** represents a valid test, while **b** represents a test were verification phase maximal oxygen uptake (V̇O_2max_) was higher than that reached during incremental phase, consequently, an invalid test. Note: differences between phases were considered as V̇O_2_ differences > 2%; > 3%; > 2.2 or 2.0 ml kg^−1^ min^−1^ and V̇O_2_ values presented as 15-s averages

Finally, researchers, physiologists and clinicians should keep in mind that the primary goal of the phase is not simply to achieve a V̇O_2_ similar to the incremental phase. Rather, the goal is to create a platform that enables a higher V̇O_2_ to be reached if it has not been reached previously (Fig. 2). Thus, the intensity selected should be sufficient to generate increments (or differences) in V̇O_2_ greater than the total measurement error (this topic will be addressed in the “Data Processing” section).

#### Duration

Another important aspect that can interfere with the result of the verification phase is its duration or, more precisely, the time to exhaustion during the phase. This means that the duration of the verification phase should be sufficient to allow V̇O_2max_ to be reached or evoked. In addition, it means that the duration of the phase is closely related to the intensity employed. Recently, it has been suggested that the time to exhaustion in the intensities mentioned in the previous section is between 3 and 6 min [12]. Still, several studies reported shorter times to exhaustion during their verification phases [29, 35, 40, 42, 43, 45, 49–53]. Rossiter et al. [29] reported time to exhaustion of only 1.47 min with a mean difference between incremental and verification phase V̇O_2max_ of only 31 ml min^−1^ (i.e. less than 1%). Sawyer et al. [53] reported 1.91 min in sedentary obese and found no relationship between the duration of the verification phase and the difference between incremental test and verification phase V̇O_2max_. Furthermore, another previous study demonstrated that shorter times to exhaustion did not systematically influence the ratio between verification phase and incremental test V̇O_2peak_ in para-athletes who had been able to exercise for more than 100 s [52]. However, for those who exercised for 90 s or less, duration seemed to exert influence. Therefore, the V̇O_2_ kinetics of the population being assessed should be taken into account when planning or analysing the verification phase duration [11, 54]. That is, untrained subjects, older individuals, or patients with chronic heart failure, for example, may require a relatively greater time to achieve V̇O_2max_ compared to healthy, active, or trained subjects. Notwithstanding, considering the intensities commonly applied, this duration (i.e. ≤ 2 min) may be somewhat “tight” for certain subjects, especially if associated with very high intensities, which may result in premature fatigue, as stated in the “Intensity” section.

A possible alternative is to perform multistage rather than square-wave verification phases (Fig. 1). Thus, subjects exercise at lower intensities before the workload corresponding to the verification phase is applied [36, 40]. This prior “warm-up stage” can enhance oxygen uptake, increasing the possibility to reach V̇O_2max_ before exhaustion [37]. It was already shown that previous moderate and high-intensity exercises can increase V̇O_2_ kinetics [55, 56] and improve performance in supramaximal exercises [57]. As such, Midgley et al. [36] proposed a multistage verification phase comprising 2 min at 50% iV̇O_2max_ and 1 min at 70% followed by one stage higher than the last completed stage in the incremental test. Similarly, Scharhag-Rosenberger et al. [40] employed 1 min at 60% iV̇O_2max_ followed by 110% iV̇O_2max_ until exhaustion. Both protocols were able to confirm 90–95% of the V̇O_2max_ identified at the incremental test. Accordingly, based on studies conducted so far (Table 1), it seems plausible to suggest that durations between 2 and 3 min should be adequate to confirm V̇O_2max_ during verification phases, although some clinical populations may require longer periods. In addition, exercise mode should also be observed because subjects exercising on a cycle ergometer would be more susceptible to local muscle fatigue compared to treadmill running which, in turn, could lead to a shorter verification phase duration on the first of these (i.e. cycle ergometer).

#### Recovery—How Long to Wait?

Another factor that could possibly influence the verification phase is the time elapsed between incremental test and the verification phase. If a true maximal effort is provided by an individual during the incremental test, there will be a number of key perturbations to the skeletal muscle milieu, including depleted phosphocreatine stores coupled with increased metabolic acidosis. Performing the verification phase while inadequately recovered could lead to premature fatigue and inability to achieve a “true” V̇O_2max_. To the best of the author’s knowledge, no study to date has investigated the influence of physiological outcome recovery on the V̇O_2max_ attained during the verification phase and, accordingly, this should be kept in mind when considering the following results.

Recovery periods of 10 min were the most frequently employed, although 5, 15, 20 and 60 min were also used (Table 1). Nolan et al. [44] found no difference (i.e. V̇O_2max_ > ± 3%) comparing 20 and 60 min recovery periods, recommending that 20 min should suffice for physically active subjects. Recently, it has been suggested that for healthy individuals, shorter recovery intervals (i.e. 5 or 10 min) would already be sufficient [12]. Actually, 1- and 3-min recovery periods have already been successfully employed in this population [41, 42, 51]. Therefore, as stated by Poole and Jones [12], recovery time between phases does not appear to be critical, although shorter periods may be more time-efficient. Notwithstanding, it is still necessary to experimentally confirm this in older adults and clinical populations. In addition, research investigating the relationship between the recovery of certain physiological responses (e.g. phosphocreatine and metabolic acidosis), the time between incremental test and verification phase and the V̇O_2max_ achieved during the verification phase is also warranted.

#### Data Processing

Even if intensity, duration and recovery are adequately planned, inadequate data processing can interfere with the verification phase results and, ultimately, lead to flawed conclusions. Despite this, no study to date directly addressed the relationship between different sampling intervals and V̇O_2max_ attainment during the verification phase. Previous investigations have shown that both V̇O_2max_ and plateau incidence during an incremental test can differ according to the sampling interval employed. Specifically, Astorino et al. [22] found that 11-breath moving averages and 15-s averages of V̇O_2_ data showed higher incidence of plateau (i.e. 100%) compared to 30-s (57%) and 60-s (8%) averages. In addition, Astorino [10] demonstrated that 15- and 30-s averages, as well as breath-by-breath data, resulted in higher V̇O_2max_ values when compared to 60-s averages, which was also observed by Robergs [58]. Consequently, Robergs et al. [30] suggested that “time-averaged sampling should be no longer than 30 s” while “breath-averaged sampling should be” performed by “15-breath running averages”.

Thus, considering that the verification phase resembles an incremental discontinuous test, it could be suggested that V̇O_2_ data from verification phases are processed through 15- to 30-s time intervals, for example, although some authors advocate that 15 s averages are preferable [22, 31]. Since 15-s averages may identify the highest V̇O_2max_ [31] while also enhancing the incidence of plateaus in the incremental tests [22], it would allow researchers to use two criteria rather than just one to confirm V̇O_2max_. However, it is required that future studies address these questions and confirm whether these sampling intervals are best suited for the comparison of incremental and verification phases. Additionally, other data processing techniques such as digital filtering [30] should also be investigated.

#### Validating the Verification Phase

Professor Robert Pettitt once mentioned: “That is where art separates from science: at some point, you’ve got to pick a criterion”. As previously mentioned, a true V̇O_2max_ can be confirmed when incremental test and verification phase V̇O_2max_ values do not differ. Nevertheless, what is or should be the appropriate cutoff point to define whether these results are similar? Some previous investigations compared the mean V̇O_2max_ between the two phases and when no statistical difference was identified, V̇O_2max_ was deemed true. However, concerns have been expressed about this procedure [11, 39, 59, 60]. Specifically, V̇O_2max_ comparisons through the mean values can mask a considerable between-trials variability in a few participants, even when no difference between the mean values is identified (i.e. *P* > 0.05). Thus, the most appropriate procedure seems to be an individual approach [11, 36, 39]. Midgley et al. [35] suggested that a difference ≤ 2%, based on the equipment measurement error, would be an acceptable estimate. Notwithstanding, this criterion does not take into account V̇O_2max_ biological variability [2, 11], which may represent up to 90% of $\dot{V}{\mathrm{O}}_{2\max }$ total variability when assessed over different days [34]. Therefore, Kirkeberg et al. [41] suggested that differences < 3% were used to confirm $\dot{V}{\mathrm{O}}_{2\max }$ in recreationally trained subjects, which could vary according to sampling rate and subjects’ fitness level (see also Pettitt et al. [39]). In fact, based on the intra-subject coefficient of variation, several studies employed a 3% cutoff point with some degree of success, being able to use it to confirm incremental test V̇O_2max_ in healthy, active and trained subjects [42–44, 50, 61]. On the other hand, Saynor et al. [45] reported a 9% intra-subject coefficient of variation in children with cystic fibrosis, showing that for some patient populations, less restrictive percentages may be necessary when comparing V̇O_2max_ between incremental and verification phases. Whichever the case, if an acute measurement is to be performed, there is a possibility that biological or day-to-day variability will not represent a substantial part of V̇O_2max_ total variability as the time elapsed between incremental test and verification phase is usually short (i.e. few minutes). Consequently, short-term variability and equipment measurement error would be the main factors affecting this variability. However, if a chronic assessment is to be considered (e.g. pre-post a training intervention), short-term variability and equipment measurement error, but also day-to-day variability, may potentially influence the results. As only a few studies to date have employed the verification phase to confirm V̇O_2max_ in longitudinal studies, the validity of different criteria on this type of research remains to be tested.

Alternatively, Midgley et al. [36] proposed the utilization of the difference between the verification phase V̇O_2max_ and the V̇O_2max_ modelled from a least-square linear regression based on the linear portion of the incremental test V̇O_2_-workrate curve. Differences greater than 50% between measured and modelled values would indicate a plateau in the V̇O_2_ and confirmation of a “true” V̇O_2max_. According to the authors, the advantage of this criterion would be that it is specific to subject and test characteristics (e.g. protocol, ergometer). Notwithstanding, the 50% value was based on an arbitrary choice and, therefore, needs further investigation although the rationale seems reasonable [36]. Thus, there seems to be no consensus as to the best individual-based criterion to be used to validate verification phase V̇O_2max_. Accordingly, although the verification phase “science” is differentiating itself from “art”, criteria need to be based on the highest category of scientific evidence and, therefore, a great effort is still needed before a consensus is found. Future studies comparing different criteria may help shed some light on this topic. In addition, studies should, whenever possible, make clear what criteria were used and why they were chosen, as well as report reliability measures such as V̇O_2max_ typical errors and coefficients of variation [62]. It is also advisable that scientists should develop these criteria in their laboratories rather than using values developed by other laboratories with different equipment and methodologies.

#### Additional Considerations

To rely on a single incremental test and verification phase protocol to meet all characteristics of a wide range of populations is somewhat over simplistic or naïve. Therefore, based on a pragmatic perspective, a plausible proposal would be to structure the incremental protocol according to the variables that will be assessed (e.g. V̇O_2max_, lactate threshold, percentage of HR associated to the ventilatory threshold). Verification phase, in turn, would be designed based on the incremental test protocol, as discussed in sections “Verification Phase: a Light at the End of the Tunnel?” and “How It Should Be Done—Is There Consensus?”. Accordingly, it is important that authors clearly present their reasons for choosing that specific verification phase. Moreover, one major advantage of the verification phase is that it deals with the same unit as the incremental test (i.e. l min^−1^ or ml kg^−1^ min^−1^). Thus, when a higher V̇O_2max_ is identified in the verification phase and it is not possible to perform a new incremental test and/or verification phase, for example, researchers are provided with the highest V̇O_2_ value reached, which would not be possible through other criteria based solely on a single incremental test (Fig. 2b).

In fact, this raises an important methodological question: “What actually is V̇O_2max_ after performing the incremental test and the verification phase?”. The majority of studies to date found both incremental test and verification phase V̇O_2max_ to be similar (Table 1), but did not present a clear suggestion as to which value was to be selected. Possibilities are that the V̇O_2max_ value from the incremental test, the verification phase (possibly the higher) or the averaged value from both may be selected. Because both phases need to present sufficiently similar V̇O_2max_ results to validate the test (as explained in section “Validating the Verification Phase”), it may be plausible to suggest that there should be no substantial interference on V̇O_2max_ results irrespective of which V̇O_2max_ value is selected, although this should be clearly stated in the manuscript. Finally, it may also be possible for a subject to terminate the two phases at sub-maximal efforts resulting in similar V̇O_2max_ values in both phases. Even though it may happen, it is unlikely to occur, especially if the verification phase is performed with an adequate duration (see the “Duration” section). Actually, this is exactly what favors the verification phase as it requires this submaximal value to happen twice and not only once as in an ordinary incremental test.

### Is It Safe?

Because subjects need to perform two efforts to the point of exhaustion, researchers may worry about possible complications arriving from the extra effort. When considering all studies presented in Table 1, a total of approximately 834 subjects comprising healthy children, adults and elderly, athletes, overweight/obese and patient populations were assessed. Hawkins et al. [32] performed a total of 156 verification phases at 130% iV̇O_2max_ in well-trained runners. According to the authors, no adverse events occurred during verification phases. Nevertheless, it may be suggested that as the subjects performed the verification phase the next day, this result should not be extrapolated to phases performed in the same session. In this regard, physically active subjects and athletes submitted to a verification phase 1 and 3 min after the incremental test, respectively, also did not present any complication [51]. This result is also corroborated by other studies [41, 42], including sedentary and untrained subjects [31, 40] and also children [49].

Recently, the possibility that, at least in a clinical setting, the verification phase would be “unrealistic and unethical in certain patient populations” was raised [63]. When only those studies that assessed special populations are taken into account (Table 1), out of ~ 241 subjects evaluated, only three cases related to the verification phase were reported. One obese subject and another with chronic heart failure requested the verification phase not to be performed [53, 64], whereas only one chronic heart failure patient did not perform his verification phase due to the “onset of runs of multifocal ectopic beats” [53]. Thus, provided that all safety measures are taken, the verification phase seems to be well tolerated in both healthy and patient populations in general [12, 65].

### Future Directions

It is clear that the verification phase can be a practical and sensitive method to confirm a maximal incremental test V̇O_2max_. However, there are still some issues that deserve to be addressed. Specifically, to the best of the author’s knowledge, no study has sought to identify a verification protocol that is feasible for different populations (e.g. healthy adults and elderly). Such a protocol could allow a better comparison of verification phase V̇O_2max_ across different studies, and an adequate comparison of other outcomes assessed as well (e.g. maximal power output, ventilatory thresholds). It could also assist researchers to investigate the effects of an outcome on different populations’ V̇O_2max_. As an example, the effect of aging on V̇O_2max_ can be studied based on a protocol that is feasible for both adults and elderly subjects and that does not rely on secondary criteria or peak V̇O_2_.

Furthermore, comparisons between square-wave and multistage verification protocols can also enhance knowledge on the applicability of these models, especially in patient populations who could benefit from the possibility to enhance their $\dot{V}{\mathrm{O}}_2$ kinetics and time to exhaustion. Additionally, current criteria used to compare the verification phase and incremental test V̇O_2max_ still warrant further investigation. Likewise, as V̇O_2max_ should be confirmed on an individual basis, mean value comparisons between submaximal and supramaximal verification phases may not be the ideal approach and future studies may seek to examine and compare the differences between these protocols based on individual differences.

Recently, Astorino et al. [66] compared V̇O_2max_ after 20 sessions of high-intensity interval training in three different groups. The authors reported that V̇O_2max_ was enhanced between 8.9 and 12.3% in the three groups and that results from the verification phases suggested “that participants did exhibit ‘true’ V̇O_2max_ and that” their “reported increases in V̇O_2max_ are repeatable and not due to random error”. Therefore, experimental studies employing verification phases are also warranted in order to identify if it can provide any further information or how does it impact the outcomes compared to the incremental test alone. Last but not least, investigations aimed at assessing the verification phase as well as those experiments employing it should consider using the keyword “verification phase” to help researchers retrieve these studies more easily.

## Conclusions

Although the plateau is still considered the best evidence to confirm V̇O_2max_, its frequency is or may be low. Moreover, secondary criteria, at least those mentioned in the present review, lack sensitivity and reliability to confirm V̇O_2max_, which lead authors to suggest its complete rejection. On the other hand, as discussed throughout this review, the verification phase was demonstrated as a practical and sensitive method to confirm V̇O_2max_ among different populations. Accordingly, both supramaximal and submaximal protocols appear to be suitable as long as the incremental test design is taken in consideration, while verification phase durations between 2 and 3 min should already be adequate for most, but not all, subjects. As suggested by previous researchers, recovery between incremental and verification phases does not appear to be critical, although this recommendation still requires further investigation in older adults and clinical populations. Further, despite not being addressed directly during verification phases, processing data with 15- to 30-s averages seems reasonable, while 15-s averages may also enhance plateau incidences. Additionally, incremental and verification phase V̇O_2_ comparisons should be performed on an individual basis and not based on group means comparisons. In this regard, whenever possible researchers should provide reliability measures such as coefficients of variation and typical errors. Finally, as already mentioned, researchers, physiologists and clinicians must keep in mind that the main purpose of the verification phase is not simply to achieve a V̇O_2_ similar to the incremental phase, but to create a platform that enables a higher V̇O_2_ to be reached if it has not been reached previously.

## Additional File

Additional file 1: Table S1. Incidence of V̇O_2max_ deemed valid based on the verification phase as well as phase duration and method employed for comparison between phases along other criteria employed. (PDF 316 kb)
