# Supplementary material for: The Maximal Oxygen Uptake Verification Phase: a Light at the End of the Tunnel?
Source: Sports Med Open. 2017 Dec 8;3:44. doi: 10.1186/s40798-017-0112-1 (PMC5721097; doi:10.1186/s40798-017-0112-1)
Supplement: Additional file 1: Table S1. — Incidence of V̇O2max deemed valid based on the verification phase as well as phase duration and method employed for comparison between phases along other criteria employed. (PDF 316 kb) [file 40798_2017_112_MOESM1_ESM.pdf]

Table S1. Incidence of $\dot{V}O_{2max}$ deemed valid based on the verification phase as well as phase duration and method employed for comparison between phases along other criteria employed.

Reference	*n*	Training status	Percentage of subjects who achieved criteria						Phase duration	Comparison between incremental and verification phase $\dot{V}O_{2max}$
			$\dot{V}O_2$ plateau	RER ≥ 1.15	RER ≥ 1.10	HR (220-age)	[LAC]	Verification phase		
Niemelä et al. [16]	16 men	Not reported	12.5%	37.5%	-	31.3%	-	56.3%	~13.1 min; 13.3 min	Mean
Stachenfeld et al. [67]	33 men 18 women	Not reported	88.0%	-	-	-	-	88.0%	Not reported	Mean; difference ≤ 150 $ml.min^{-1}$ between phases
Day et al. [28]	71 men	Not reported	17.0%	-	-	-	-	100%	~4-10 min	Mean
Midgley et al. [35]	16 men	Long- and middle-distance runners	50.0%	-	81.3%	81.3%	-	87.5%	~2.8 min	Difference ≤ 2% between phases*
	16 men		50.0%	-	68.8%	75.0%	-	75.0%	~2.9 min	Difference ≤ 2% between phases*
Rossiter et al. [29]	7 men	Not reported	0%	~50.0%	-	~100%	-	100%	~1.5 min	Mean
		Not reported	0%	~50.0%	-	~100%	-	100%	~2.2 min	Mean
Midgley et al. [37]	9 men	Distance runners	-	-	-	-	-	~100%	Not reported	Mean
			-	-	-	-	-	~100%	Not reported	Mean
			-	-	-	-	-	~100%	Not reported	Mean
Hawkins et al. [32]	52 men	Well-trained distance runners	-	-	-	-	-	100%	at least 2.3 min	Mean*

Foster et al. [51]	16 men 4 women	Physically active	-	-	-	-	-	100%	~1.5-3 min	Mean
	12 men 8 women	Cross-country trackers and triathletes	-	-	-	-	-	100%	~1.5-3 min	Mean
Poole et al. [17]	8 men	Recreationally trained	62.5%	87.5%	87.5%	62.5%	25.0%	100%	Not reported	Mean*
Astorino et al. [31]	6 men 9 women	Sedentary	-	~100%	~100%	-	-	87.0%	~4.2 min	Mean
	1 men 8 women	Sedentary	-	~100%	~100%	-	-	67.0%	~3.7 min	
Midgley et al. [36]	10 men	Cyclists	80.0%	100%	100%	90.0%	90.0%	90.0%	~4.7 min	Difference > 50% between measured and modelled verification phase $\dot{V}O_{2max}$
	10 men	Runners	70.0%	40.0%	90.0%	80.0%	40.0%	90.0%	~4.5 min	
Astorino & White [61]	13 men 17 women	Physically active	-	-	100%	-	-	76.7%	~2.1 min	Mean; difference ≤ 2% between phases*
Wood et al. [68]	67 men 68 women	Sedentary overweight and obese	46.0%	89.0%	-	83.0%	70.0%	84.4%	Not reported	Difference < 50% between incremental and verification phases $\dot{V}O_{2max}$ based on ACSM equation
Kirkeberg et al. [41]	12 men	Recreationally trained	-	-	58.3%	92.0%	-	100%	~2.8 min	Mean*
			-	-	75.0%	92.0%	-	100%	~3.1 min	
			-	-	25.0%	92.0%	-	100%	~3.5 min	
Scharhag-Rosenberger et al. [40]	20 men 20 women	Not reported	30.0%	-	20.0%	76.0%	78.0%	95.0%	~2.1 min; ~2.4 min	Mean; difference ≤ 5.5% between phases*

Bowen et al. [64]	24 men	Chronic heart failure patients	-	-	75.0%	-	-	58.0%	~2 min	Mean; individual comparison with non-paired *t*-test
Beltrami et al. [69]	23 men<br>3 women	Cross-country skiers and runners	69.2%	-	-	-	-	92.3%	Not reported	Mean; difference ≤ 123±18 $ml.min^{-1}$ O_2 between stages*
Leicht et al. [52]	8 men	Wheelchair Rugby Players	31.3%	75.0%	81.3%	-	44.0%	100%	~1-3.7 min	Mean*
	8 men	Wheelchair Basketball Players	37.5%	100%	100%	87.5%	93.8%	100%		
	8 men	Wheelchair Basketball and Tennis Players	50.0%	75.0%	93.8%	43.8%	93.8%	100%		
Mier et al. [24]	8 men 27 women	College athletes	14.0%	69.0%	91.0%	83.0%	-	60-80%	at least 4 min	Difference ≤ 2.2 and ≤ 2 $ml.kg^{-1}.min^{-1}$ O_2
Dalleck et al. [50]	9 men<br>9 women	Middle-aged and Older Adults	-	-	-	-	-	88.9%	~2.6 min	Mean; difference ≤ 3% between phases*
Saynor et al. [45]	10 men<br>4 women	Cystic fibrosis patients	7.1%	100%	100%	21.2%	71.4%	79.0%	~1.2 min	Mean; difference ≤ 9% between phases
Sedgeman et al. [42]	6 men<br>7 women	Recreationally trained	100%	-	-	-	-	76.9%	~2.2 min	Mean; difference ≤ 3% between phases*
			69.2%	-	-	-	-	76.9%	~1.3 min	
Nolan et al. [44]	6 men<br>7 women	Physically active	-	-	-	-	-	100%	~5.3 min	Mean; difference ≤ 3% between phases*
			-	-	-	-	-	100%	~5.2 min	

			-	-	-	-	-	66.7%	~4.5 min	
			-	-	-	-	-	58.3%	~4.4 min	
Straub et al. [48]	12 men<br>4 women	Trained cyclists	18.8%	-	37.5%	37.5%	87.5%	94.0%	Not reported	Difference ≤ 150 $ml.min^{-1}$ O_2
Sawyer et al. [53]	10 men<br>9 women	Sedentary obese	-	-	-	-	-	31.6%	~1.9 min	Mean; difference greater than 2% between phases*
Scheadler & Devor [70]	13 subjects	Trained endurance runners	-	-	-	-	-	92.3%	~2.5 min	Difference ≤ 50 $ml.min^{-1}$ between phases
Colakoglu et al. [71]	9 men	Mildly- to well-trained cyclists and track and field athletes	-	-	-	-	-	0%	~4.6 min	Mean
Taylor et al. [72]	11 men<br>8 women	Runners and Triathletes	-	-	-	-	-	100%	~3 min	Difference ≤ 2% between phases*
Weatherwax et al. [43]	18 men<br>6 women	Elite endurance athletes	-	-	-	-	-	100%	~2.8 min	Mean; difference ≤ 3% between phases*
Bhammar et al. [49]	9 boys<br>3 girls	Obese	44.4%	-	77.8%	33.0%	-	22.0%	~2.3 min	Mean; validated when difference between measured phases' $\dot{V}O_2$ was lower than the predicted difference between phases.
	5 boys<br>6 girls	Non-obese	33.0%	-	88.9%	44.4%	-	44.4%	~2.1 min	

$\dot{V}O_2$, oxygen uptake; $\dot{V}O_{2max}$, maximal oxygen uptake; RER, respiratory exchange ratio; HR, heart rate; [LAC], blood lactate concentration; * indicates that reliability measures such as typical error, coefficient of variation or intraclass correlation coefficient were provided. Note: whenever possible, authors were contacted to provide missing data.
